# National clinical and financial outcomes associated with acute kidney injury following esophagectomy for cancer

**DOI:** 10.1371/journal.pone.0300876

**Published:** 2024-03-28

**Authors:** Ayesha P. Ng, Nikhil Chervu, Corynn Branche, Syed Shahyan Bakhtiyar, Mehrab Marzban, Paul A. Toste, Peyman Benharash

**Affiliations:** 1 Cardiovascular Outcomes Research Laboratories, David Geffen School of Medicine at UCLA, Los Angeles, California, United States of America; 2 Department of Surgery, David Geffen School of Medicine at UCLA, Los Angeles, California, United States of America; 3 Department of Surgery, University of Colorado Anschutz Medical Center, Aurora, Colorado, United States of America; Stanford University School of Medicine, UNITED STATES

## Abstract

**Background:**

Esophagectomy is a complex oncologic operation associated with high rates of postoperative complications. While respiratory and septic complications have been well-defined, the implications of acute kidney injury (AKI) remain unclear. Using a nationally representative database, we aimed to characterize the association of AKI with mortality, resource use, and 30-day readmission.

**Methods:**

All adults undergoing elective esophagectomy with a diagnosis of esophageal or gastric cancer were identified in the 2010–2019 Nationwide Readmissions Database. Study cohorts were stratified based on presence of AKI. Multivariable regressions and Royston-Parmar survival analysis were used to evaluate the independent association between AKI and outcomes of interest.

**Results:**

Of an estimated 40,438 patients, 3,210 (7.9%) developed AKI. Over the 10-year study period, the incidence of AKI increased from 6.4% to 9.7%. Prior radiation/chemotherapy and minimally invasive operations were associated with reduced odds of AKI, whereas public insurance coverage and concurrent infectious and respiratory complications had greater risk of AKI. After risk adjustment, AKI remained independently associated with greater odds of in-hospital mortality (AOR: 4.59, 95% CI: 3.62–5.83) and had significantly increased attributable costs ($112,000 vs $54,000) and length of stay (25.7 vs 13.3 days) compared to patients without AKI. Furthermore, AKI demonstrated significantly increased hazard of 30-day readmission (hazard ratio: 1.16, 95% CI: 1.01–1.32).

**Conclusions:**

AKI after esophagectomy is associated with greater risk of mortality, hospitalization costs, and 30-day readmission. Given the significant adverse consequences of AKI, careful perioperative management to mitigate this complication may improve quality of esophageal surgical care at the national level.

## Introduction

Surgical resection, with or without neoadjuvant chemoradiotherapy, is a mainstay of treatment for esophageal and gastroesophageal junction cancers [1]. Significant advances in surveillance programs, perioperative management, and multimodal treatment regimens have helped improve postoperative outcomes, with 5-year survival nearing 50% [2]. Despite such progress, esophagectomy remains a high-risk procedure regardless of operative approach with reported complication rates ranging from 40–60% [3, 4]. Importantly, pneumonia, anastomotic leak, and infectious complications after esophagectomy have been linked to early cancer recurrence and reduced long-term survival [5, 6].

More recently, acute kidney injury (AKI) and associated outcomes have garnered significant attention as an adverse event across many operations [7–9]. In the context of esophagectomy, a single-center study of 898 patients in the US reported an incidence of 11.9%, whereas a multi-center study of 1,135 patients in the UK and Ireland reported 18.3% of patients with postoperative AKI [10, 11]. Age, preoperative renal insufficiency, operative time, and perioperative blood transfusions have all been cited as risk factors associated with AKI [10, 12, 13]. With prior studies limited in sample size and primarily focused on identifying risk factors, the impact of AKI on postoperative outcomes at the national level has yet to be characterized.

The present study characterized the incidence, risk factors, and in-hospital outcomes associated with AKI among a contemporary national cohort of patients receiving esophagectomy for cancer. We hypothesized AKI to be independently associated with increased odds of index mortality, hospitalization costs, length of stay, and readmission.

## Methods

This was a cross-sectional study using the 2010–2019 Nationwide Readmissions Database (NRD). Maintained by the Healthcare Cost and Utilization Project (HCUP), the NRD is the largest publicly available all-payer readmissions database in the United States [14]. Hospital discharge data in the NRD are collected from individual State Inpatient Databases, which contain deidentified unique patient linkage numbers used to track patients across hospitals within a state. Each sampled institution has assigned discharge weights allowing for survey-weighted national estimates of 36 million discharges each year, representing approximately 60% of all hospitalizations in the United States [14]. All elective adult hospitalizations (≥18 years) for esophagectomy with a diagnosis of esophageal or gastric cancer were identified using relevant *International Classification of Diseases 9*^*th*^*/10*^*th*^ *Revision* (ICD-9/10) diagnosis and procedure codes (S1 Table). Patients were stratified into the *AKI* group if specific diagnostic codes for acute kidney injury (584, N17) were present (otherwise *no-AKI*). Patients with history of end-stage renal disease or chronic dialysis dependence were excluded.

Patient and hospital characteristics including age, sex, income quartile, primary payer, and hospital teaching status were defined using the HCUP Data Dictionary [14]. History of radiation/chemotherapy and comorbidities such as diabetes, hypertension, chronic kidney disease stages 1–5, lung disease, liver disease, congestive heart failure, pulmonary circulation disorders, and neurologic disorders were identified using ICD-9/10 diagnosis codes (S1 Table). The Elixhauser Comorbidity Index, a validated composite of 30 comorbidities, was additionally used to quantify the overall burden of chronic conditions at index admission [15]. ICD-9/10 procedure codes were used to ascertain open and minimally invasive (MIS), including laparoscopic and robotic, surgical approaches as well as requirement of renal replacement therapy (S1 Table). Hospitals were stratified into low, medium, and high volume tertiles based on annual institutional case volume of esophagectomy. Perioperative complications included cerebrovascular (stroke), thromboembolic (deep vein thrombosis, pulmonary embolism), cardiac (cardiac arrest, myocardial infarction), pulmonary (respiratory failure, prolonged mechanical ventilation, pneumonia), infectious (septicemia, abscess, wound infection), and intraoperative (hemorrhage, accidental puncture, phrenic or vagus nerve injury) complications, as well as requirement of blood transfusion (S1 Table). The Clavien–Dindo classification system was used to classify the severity of postoperative complications as no complication/grade I, grade II, grade III, and grade IV/V, according to the ICD algorithm developed by Lentine et al. [16]. Hospitalization costs were calculated from charges using hospital-specific cost-to-charge ratios and were inflation adjusted to the 2019 Patient Health Care Index [17]. The primary outcome of interest was in-hospital mortality, while secondary outcomes included index length of stay (LOS), hospitalization cost, non-home discharge, and 30-day nonelective readmission.

Categorical variables are reported as frequencies (%) and compared using the Pearson’s chi-square test. Continuous variables are reported as means with standard deviation (SD) or medians with interquartile range (IQR) and compared using the adjusted Wald or Mann-Whitney U tests, respectively. Significance of temporal trends was assessed using Cuzick’s nonparametric test for trends (nptrend) [18]. Multivariable linear and logistic regression models were developed to identify risk factors for AKI and assess its independent association with outcomes of interest. Variable selection was performed by applying the Least Absolute Shrinkage and Selection Operator (LASSO) to enhance model generalizability and minimize overfitting and collinearity between independent variables [19]. Models were evaluated using the receiver operating characteristics curve as well as Akaike and Bayesian information criteria.

The cumulative risk of nonelective readmission within 30 days of index discharge was evaluated using Royston-Parmar’s flexible parametric regression [20]. This methodology allows for varying hazards of readmission over time and accounts for differences in patient, operative, and hospital characteristics between groups. The hazards were calculated over time to readmission using 2 restricted cubic spline knots. Regression results are reported as adjusted odds ratios (AOR) for dichotomous outcomes and beta coefficients (β) for continuous variables with 95% confidence intervals (CI). The Stata “margins” command was used to predict absolute risk-adjusted values for costs and LOS based on the output of relevant regressions. Statistical significance for all analyses was set at α = 0.05. All statistical analyses were performed using Stata 16.1 (StataCorp, College Station, TX). This study was deemed exempt from full review by the Institutional Review Board at the University of California, Los Angeles due to the de-identified nature of the NRD (accessed July 18, 2022).

## Results

**Fig 1:**
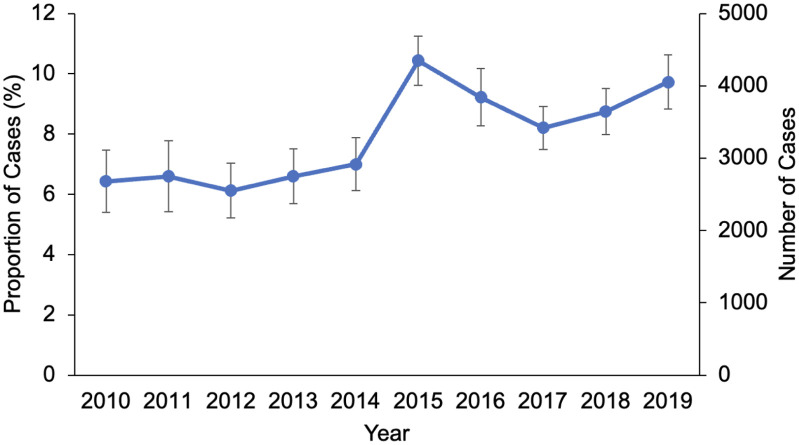
Temporal trends in incidence of acute kidney injury after esophagectomy for cancer. *Nptrend < 0*.*001*.

Of an estimated 40,438 cancer patients undergoing esophagectomy, 3,210 (7.9%) developed AKI. Among patients with AKI, 5.7% required renal replacement therapy. Over the 10-year study period, the incidence of AKI increased from 6.4% to 9.7% (nptrend<0.001, Fig 1). On examination of concurrent temporal trends that may help explain the increasing AKI incidence, we found that the average age, Elixhauser Comorbidity Index and prevalence of diabetes and chronic kidney disease also significantly increased over the study period (nptrend<0.001). In addition, the proportion of patients with prior chemoradiation therapy increased significantly from 17.3% in 2010 to 38.5% in 2019 (nptrend < 0.001), while patients with MIS operations also increased from 6.6% to 39.2% (nptrend < 0.001). Compared to *nAKI*, *AKI* patients were older (66 ± 9 vs 64 ± 10 years, p<0.001) and had a higher burden of comorbidities (Elixhauser Index 4.1 ± 1.5 vs 3.6 ± 1.5, p<0.001, Table 1). Patients with AKI were less commonly female (12.7 vs 18.3%, p<0.001) and less often had prior radiation/chemotherapy (14.0 vs 32.2%, p<0.001). In addition, the *AKI* cohort less frequently had private insurance (31.1 vs 42.9%, p<0.001) or robotic-assisted operations (5.5 vs 9.1%, p<0.001) relative to the *nAKI* cohort. Hospitals in the *AKI* group had significantly lower annual institutional volume of esophagectomy compared to *nAKI* hospitals (26 [IQR: 10–59] vs 34 [13–70], p<0.001).

**Table 1:** Patient, operative, and hospital characteristics stratified by incidence of acute kidney injury (AKI) after esophagectomy for cancer. *SD*: *Standard deviation*.

	No AKI (n = 37,228)	AKI (n = 3,210)	p-value
Age (years, mean, SD)	64 ± 10	66 ± 9	<0.001
Female sex (%)	18.3	12.7	<0.001
*Payer Status (%)*			<0.001
Private	42.9	31.1	
Medicare	47.0	58.3	
Medicaid	6.7	6.5	
Other	3.4	4.1	
*Income Quartile (%)*			0.04
Fourth (highest)	25.3	22.5	
Third	27.3	25.6	
Second	26.6	29.5	
First (lowest)	20.7	22.4	
*Cancer Type (%)*			0.02
Esophageal cancer	64.4	67.9	
Gastric cancer	35.5	32.1	
History of radiation/chemotherapy	32.2	14.0	<0.001
*Comorbidities (%)*			
Elixhauser Index (mean, SD)	3.6 ± 1.5	4.1 ± 1.5	<0.001
Diabetes	20.6	25.2	<0.001
Hypertension	55.3	57.3	0.23
Chronic kidney disease stages 1–3	1.5	4.8	<0.001
Chronic kidney disease stages 4–5	0.2	1.7	<0.001
Chronic lung disease	20.4	24.0	0.005
Chronic liver disease	5.0	8.8	<0.001
Congestive heart failure	4.8	11.5	<0.001
Pulmonary circulation disorders	2.5	4.7	<0.001
Neurologic disorders	3.2	11.0	<0.001
*Operative Approach (%)*			<0.001
Open	76.6	80.0	
Laparoscopic	14.3	14.6	
Robotic	9.1	5.5	
Hospital Esophagectomy Volume (cases per year, median, IQR)	34 [13–70]	26 [10–59]	<0.001
*Hospital Teaching Status (%)*			0.07
Non-metropolitan	1.0	0.7	
Metropolitan non-teaching	7.9	9.8	
Metropolitan teaching	91.1	90.0	

**Fig 2:**
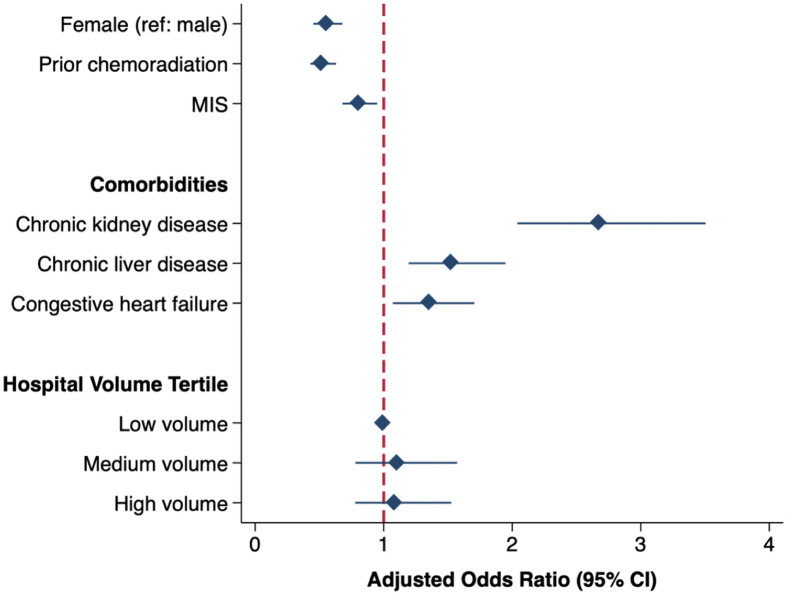
Patient, operative, and hospital characteristics associated with acute kidney injury after esophagectomy for cancer. Model C-statistic: 0.79. *Ref*: *Reference*. *CI*: *Confidence interval*. *Minimally invasive surgery (MIS) includes the laparoscopic and robotic approaches*.

Following multivariable risk adjustment, female sex was associated with significantly decreased odds of AKI (AOR 0.55 [95% CI 0.45–0.68], Fig 2, S2 Table). Comorbidities including chronic liver disease (1.52 [1.19–1.94]) and congestive heart failure (1.34 [1.06–1.69]) were associated with increased odds of AKI. In particular, preoperative chronic kidney disease (2.67 [2.04–3.50]) was a significant risk factor for AKI. Furthermore, patients with history of chemoradiation therapy (0.52 [0.43–0.63]) and MIS operations (0.80 [0.68–0.95]) had significantly reduced odds of AKI. Of note, hospital esophagectomy volume had no significant association with AKI.

Unadjusted clinical and financial outcomes are shown in Table 2. Compared to *nAKI*, the *AKI* cohort exhibited significantly increased rates of in-hospital mortality (21.7 vs 2.2%, p<0.001). Concurrent complications including infectious (43.1 vs 11.7%, p<0.001) and respiratory (57.8 vs 25.2%, p<0.001) events were also more common among patients with AKI. The *AKI* group had a significantly greater proportion of patients with postoperative complication severity grading of Clavien-Dindo Grade IV/V (55.9 vs 20.4%, p<0.001) compared to *nAKI*. Furthermore, the *AKI* group experienced significantly greater LOS (19 vs 10 days, p<0.001) and index hospitalization costs ($83,600 vs 43,900, p<0.001) relative to *nAKI*. Of note, non-home discharge (49.7 vs 14.9%, p<0.001) and 30-day nonelective readmission (16.1 vs 13.7%, p = 0.03) were significantly more common among individuals with AKI. Total costs including the index hospitalization and all readmission costs within 30 days remained greater among *AKI* patients ($95,500 vs 54,500, p<0.001) compared to *nAKI*.

**Table 2:** Clinical and financial outcomes following esophagectomy for cancer stratified by incidence of acute kidney injury (AKI). *IQR*: *Interquartile range*.

Outcome	No AKI (n = 37,228)	AKI (n = 3,210)	p-value
In-hospital mortality (%)	2.2	21.7	<0.001
*Clavien-Dindo classification (%)*			<0.001
No complication / Grade I	17.3	6.3	
Grade II	4.6	1.0	
Grade III	57.7	36.8	
Grade IV / V	20.4	55.9	
*Complications (%)*			
Cerebrovascular	0.3	1.1	<0.001
Thromboembolic	2.4	5.0	<0.001
Cardiac	2.9	9.9	<0.001
Respiratory	25.2	57.8	<0.001
Infectious	11.7	43.1	<0.001
Intraoperative	3.3	4.2	0.11
Blood transfusion	12.9	18.4	0.002
Length of stay (days, median, IQR)	10 [8–14]	19 [11–34]	<0.001
Index cost ($1000s, median, IQR)	43.9 [31.9–63.8]	83.6 [53.1–145.6]	<0.001
Non-home discharge (%)	14.9	49.7	<0.001
30-day nonelective readmission (%)	13.7	16.1	0.03

**Fig 3:**
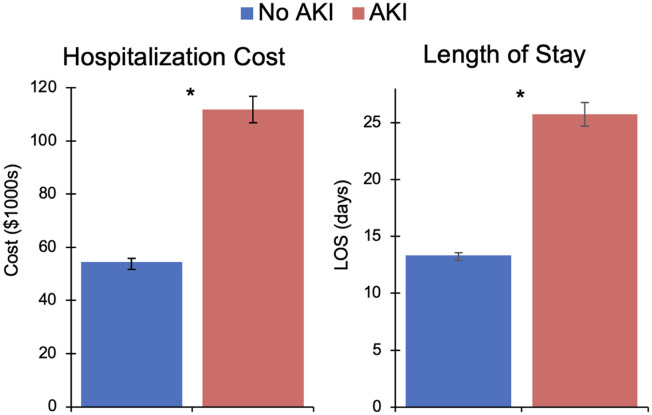
Risk-adjusted costs and length of stay (LOS) at index hospitalization associated with acute kidney injury (AKI) after esophagectomy for cancer. *p<0.001.

**Fig 4:**
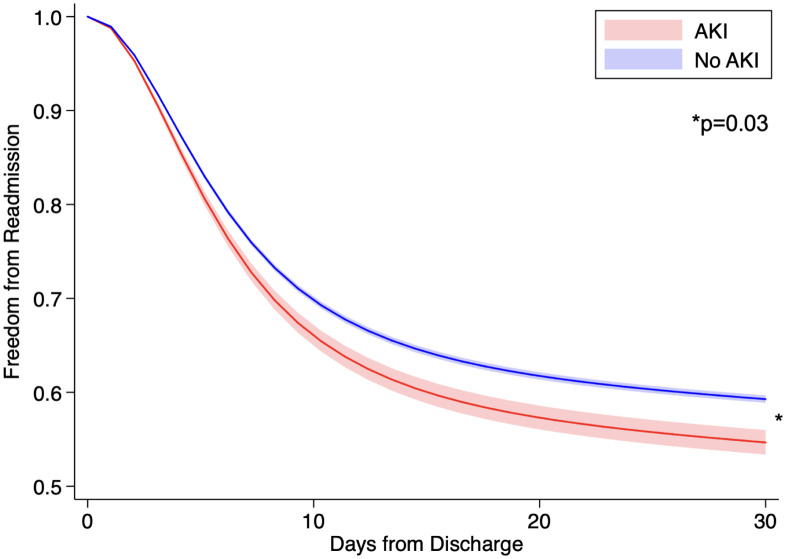
Royston-Parmar risk-adjusted hazard analysis for 30-day readmission by incidence of acute kidney injury (AKI).

On multivariable analysis, perioperative infectious (AOR: 3.82 [95% CI: 3.32–4.40]) and respiratory (2.50 [2.16–2.89]) complications were significantly associated with AKI (S2 Table). After adjustment for concurrent complications as well as key patient, operative, and hospital characteristics, AKI remained independently associated with over 4-fold greater odds of mortality (AOR: 4.61 [95% CI: 3.64–5.85]) and nearly 3-fold greater odds of non-home discharge (2.68 [2.29–3.14]). Furthermore, the *AKI* cohort incurred significantly increased attributable costs ($112,000 [107,000–117,000] vs $54,000 [53,000–56,000]) and LOS (25.7 days [24.7–26.8] vs 13.3 [13.1–13.6], Fig 3) at index hospitalization compared to the *nAKI* cohort. Of note, development of AKI was associated with 16% increased odds of 30-day nonelective readmission (AOR: 1.16 [95% CI: 1.01–1.32]). These findings were confirmed on Royston-Parmar analysis, which demonstrated significantly increased hazard of readmission within 30 days among the *AKI* cohort compared to *nAKI* (Fig 4, S3 Table).

## Discussion

Using a nationally representative cohort of patients undergoing esophagectomy for cancer, the present study evaluated clinical and financial outcomes associated with the development of perioperative AKI. Over the 10-year study period, the incidence of AKI following esophagectomy increased from 6.4% to 9.7%. Risk factors for AKI included public insurance coverage and chronic kidney disease, while prior radiation/chemotherapy and MIS operative approaches were associated with reduced odds of AKI. Notably, patients with AKI had significantly increased odds of in-hospital mortality, non-home discharge, and 30-day nonelective readmission compared to patients without AKI. In addition, AKI was associated with greater hospitalization costs and LOS. Several of these findings warrant further discussion.

The overall incidence of AKI (7.9%) was lower than reported in previous multi-center studies (12–18%), potentially due to underreporting in an administrative database [10, 11]. Comparisons with prior literature are limited by variations in definitions for AKI, surgical approach and patient risk factors. Nevertheless, AKI remains a deleterious complication that is highly predictive for mortality and resource use following esophagectomy. Of note, the present study observed increasing rates of AKI over the past decade, affecting nearly 10% of esophagectomy patients in 2019. Surgical quality improvement efforts have generally resulted in decreased mortality and complications after most types of operations. Yet AKI in particular has also been noted to be increasing in incidence after emergency abdominal operations [8]. Interestingly, we found that the average age and burden of comorbidities including chronic kidney disease among esophagectomy patients also significantly increased over the study period. Given the independent association of age and comorbidities with AKI, the older and more frail patient population with worsening baseline renal function in recent years may be responsible for the increase in incidence of AKI [21]. Recent advancements in diagnosing AKI during perioperative care may also contribute to the increase in incidence. While creatinine levels are often confounded by hemodilution perioperatively, promising novel biomarkers including cystatin C, neutrophil gelatinase-associated lipocalin, and kidney injury molecule-1 highlight early signs of renal stress before any deterioration in function and are specific to renal injury [22]. Further efforts to incorporate novel technology and standardize diagnosis of AKI in the perioperative setting may help mitigate patient morbidity.

The present study identified several risk factors associated with AKI. Consistent with prior literature, chronic comorbidities including diabetes, congestive heart failure, and preexisting kidney disease were more common among AKI patients [10, 11]. This finding suggests that lack of access to comprehensive primary and preventive care may be contributing to increased risk of AKI. In addition, neoadjuvant chemoradiation therapy was associated with decreased odds of AKI, similar to a prior national study of 1,446 esophagectomy patients with prior chemoradiation demonstrating reduced septic and renal complications [23]. Interestingly, we found that the use of neoadjuvant therapy significantly increased over time, while the incidence of AKI also increased. Chemotherapy-induced nephrotoxicity and worse baseline renal function may explain this observation [24]. However, after adjustment for comorbidities including preexisting renal failure, the independent association of chemoradiation with decreased AKI may ultimately reflect the beneficial impact of neoadjuvant therapy. Careful consideration of individual patient risk factors and improved access to comprehensive cancer centers may help guide provision of chemoradiation and prevent perioperative renal injury. Furthermore, MIS operations demonstrated significantly reduced odds of AKI following esophagectomy, which has similarly been reported in several prior studies [25, 26]. MIS approaches may lead to decreased blood loss, fluid shifts, and risk for renal injury compared to open esophagectomy approaches [27]. The persistent incidence of AKI despite the increasing use of MIS over time suggests that access to financial capital and experienced MIS esophagectomy centers may potentially be contributing to disparities in perioperative AKI. In addition, the increasing age and burden of comorbidities among esophagectomy patients over time may reflect the expansion of MIS approaches, allowing for operations on more frail patients but potentially leading to greater incidence of AKI as well.

As expected, several perioperative complications including sepsis, pneumonia, and respiratory failure were associated with AKI. AKI patients also had significantly increased severity of complications as assessed using the Clavien-Dindo classification. Of note, AKI likely triggers the development of multiple non-renal complications that collectively deteriorate a patient’s condition, which becomes challenging to quantify [11, 28]. The Comprehensive Complication Index (CCI), a weighted algorithm that adjusts for both the number and severity of complications, has previously been used in randomized trials to quantify the overall morbidity as opposed to the burden of individual complications on the patient [29, 30]. While the CCI has not yet been validated in administrative databases, incorporation of such an index into future standardized data collection for surgical complications may be warranted to further understand the multiplicative impact of complications on the risk of death [31]. The pathophysiology underlying AKI after esophagectomy is likely multifactorial. Major intraoperative fluid shifts, ischemic reperfusion events, the use of nephrotoxic drugs, and marked systemic inflammation often induced by surgical trauma may contribute to renal tubular injury [32, 33]. Goal-directed therapy (GDT) has been suggested to decrease the risk of postoperative renal injury through perioperative hemodynamic monitoring and combination of fluids and inotropes to reach adequate cardiac output (CO) and oxygen delivery (DO2) [34]. These findings are supported by several systematic reviews of randomized controlled trials, and recent guidelines by the Kidney Disease Improving Global Outcomes (KDIGO) group provide strength of recommendation 2C for GDT in prevention of perioperative AKI [34–37]. However, interventions to optimize hemodynamics remain widely variable in targets, timing, design, and technology. Standardized algorithms to guide fluid resuscitation and interdisciplinary care coordination between anesthetic and surgical teams may help mitigate AKI following esophagectomy [38].

Independent of other perioperative complications, AKI was associated with over 4-fold greater odds of in-hospital mortality as well as 16% increased odds of 30-day readmission. This study of esophagectomy patients adds to a growing body of literature demonstrating an association between even small postoperative changes in serum creatinine and worse outcomes [39–41]. While we observed a stark difference in mortality, readmissions were only moderately increased in the presence of AKI. Only 5.7% of AKI patients required renal replacement therapy, suggesting that renal injury may generally be self-limited after esophagectomy. In a recent UK study of 1,135 patients undergoing esophageal cancer operations, 70% of those with AKI exhibited recovery of renal function within 48 hours [11]. However, prior literature has demonstrated that patients with complete renal resolution of postoperative AKI still had an increased hazard ratio for long-term death of 1.20 (95% CI 1.10–1.31) [41]. The increased odds of 30-day unplanned readmission remains a key indicator for adverse long-term outcomes of AKI. Moreover, AKI was associated with significantly greater resource use and nearly doubled the index hospitalization costs and LOS, in addition to the costs accrued at readmission. In light of the rising incidence of AKI, these findings raise significant financial concern and further highlight the need for systemic efforts to mitigate AKI and reduce healthcare expenditure [39]. Further research on early screening and risk stratification for AKI as well as perioperative interventions to prevent organ hypoperfusion are needed.

The present study has several limitations inherent to its retrospective nature and the use of an administrative database. The ICD-9/10 diagnosis codes used to define the development of AKI in this study were not based on the AKI Network criteria or risk, injury, failure, loss of kidney function, and end-stage renal disease (RIFLE) criteria due to absence of values for serum creatinine or baseline renal function [42, 43]. In addition, the NRD lacks clinical granularity regarding cancer staging, time of cancer diagnosis, as well as intraoperative variables such as anesthesia duration and urinary output. Anastomotic leak is not specified in ICD-9/10 coding and was approximated through the presence of clinical manifestations, including postoperative infection, septicemia, or abscess as reported in prior analyses [44, 45]. Of note, prior use of the Clavien-Dindo classification system with HCUP data has been limited to abdominal and urological operations [16, 46, 47]. In addition, the Clavien-Dindo system does not reflect the overall impact of multiple complications on patient morbidity. The Comprehensive Complication Index (CCI) was unable to be assessed, as the CCI has not been validated in administrative databases such as the NRD and may be skewed by ICD-level coding of complications [29, 30]. ICD coding is often influenced by provider and center practices among participating hospitals in the NRD, and the transition from ICD-9 to ICD-10 may introduce variations in coding. Furthermore, our analysis was also limited to the duration of each admission and did not include outpatient data, thus potentially underestimating diagnosis of postoperative AKI and complications after hospital discharge. Despite these limitations, we used the largest, all-payer readmissions database and robust statistical methods to enhance the generalizability of our findings at the national level.

## Conclusions

The present study used a nationally representative database to demonstrate that AKI development after esophagectomy for cancer has increased over the past decade. Notably, AKI appears to be independently associated with greater risk of mortality, resource use, and 30-day readmission. Given the substantial clinical and financial implications, standardized reporting of AKI and careful perioperative management to improve end-organ perfusion are needed to help mitigate this pernicious complication. Particularly among high-risk cancer patients, predischarge interventions and care coordination to limit readmission warrant further investigation and may improve quality of esophageal surgical care at the national level.

## Supporting information

S1 Table Administrative *International Classification of Diseases*, *9*^*th*^ *and 10*^*th*^ *Revision* (ICD-9/10) diagnosis and procedure codes for esophagectomy, baseline patient characteristics, and in-hospital outcomes. (DOCX)

S2 Table Patient, operative, and hospital characteristics associated with acute kidney injury after esophagectomy for cancer. Model C-statistic: 0.79. *Ref*: *Reference*. *CI*: *Confidence interval*.
(DOCX)

S3 Table Royston-Parmar risk-adjusted hazard analysis for 30-day readmission. Restricted cubic spline knots = 2. CI: Confidence interval. Ref: Reference.
(DOCX)
