# Supplementary material for: National clinical and financial outcomes associated with acute kidney injury following esophagectomy for cancer
Source: PLoS One. 2024 Mar 28;19(3):e0300876. doi: 10.1371/journal.pone.0300876 (PMC10977786; doi:10.1371/journal.pone.0300876)
Supplement: S1 Table — (DOCX) [file pone.0300876.s001.docx]

Supplemental Table 1. Administrative *International Classification of Diseases, 9^th^ and 10^th^ Revision* (ICD-9/10) diagnosis and procedure codes for esophagectomy, baseline patient characteristics, and in-hospital outcomes.

|  | **ICD-9** | **ICD-10** |
| --- | --- | --- |
| Esophagectomy | 42.40, 42.41, 42.42, 42.52, 42.53, 42.54, 42.55, 42.56, 42.62, 42.63, 42.64, 42.65, 42.66 | 0DB50ZZ, 0DB54ZZ, 0DB10ZZ, 0DB14ZZ, 0DB20ZZ, 0DB24ZZ, 0DB30ZZ, 0DB34ZZ, 0DT50ZZ, 0DT54ZZ, 0DT10ZZ, 0DT14ZZ, 0DT20ZZ, 0DT24ZZ, 0DT30ZZ, 0DT34ZZ, 0DR507Z, 0DR547Z, 0DXE0Z5, 0DXE4Z5, 0DX80Z5, 0DX84Z5 |
| Laparoscopic | 54.21 | 0DB54ZZ, 0DB14ZZ, 0DB24ZZ, 0DB34ZZ, 0DT54ZZ, 0DT14ZZ, 0DT24ZZ, 0DT34ZZ, 0DR547Z, 0DXE4Z5, 0DX84Z5, |
| Robot-assisted | 17.41, 17.42, 17.43, 17.44, 17.45, 17.49 | 8E0W0CZ, 8E0W3CZ, 8E0W4CZ, 8E0W7CZ, 8E0W8CZ, 8E0WXCZ |
| Esophageal cancer | 150, 230.1, V10.03 | C15, D00.1, Z85.01 |
| Gastric cancer | 151, 230.2, 209.23, 209.25, V10.04 | C16, D00.2, Z85.02, C7A.092, C7A.094 |
| Acute kidney injury | 584 | N17 |
| Chronic kidney disease |  |  |
| Stage 1 | 58.51 | N18.1 |
| Stage 2 | 58.52 | N18.2 |
| Stage 3 | 58.53 | N18.3 |
| Stage 4 | 58.54 | N18.4 |
| Stage 5 | 58.55 | N18.5 |
| End-stage renal disease | 58.56 | N18.6 |
| Chronic dialysis dependence | V45.11 | Z99.2 |
| History of radiation or chemotherapy | V15.3, V87.41 | Z92.3, Z92.21 |
|  |  |  |
| **Comorbidities** |  |  |
| Diabetes | 250 | E10, E11, E13 |
| Hypertension | 401, 402, 403, 404, 405 | I10, I11, I12, I13, I15 |
| Lung disease | 416.8, 416.9, 506.4, 508.8, 500, 501, 502, 503, 504, 505, 490, 491, 492, 493, 494, 495, 496 | I27.8, I27.9, J68.4, J70.1, J70.3, J40, J41, J42, J43, J44, J45, J46, J47, J60, J61, J62, J63, J64, J65, J66, J67 |
| Liver disease | 070.22, 070.23, 070.32, 070.33, 070.44, 070.54, 070.6, 070.9, 456.0, 456.1, 456.2, 572.2, 572.3, 572.4, 572,8, 573.3, 573.4, 573.8, 573.9, V42.7, 570, 571 | K70, K72, K73, K74, B18, I85, K76.3, K76.4, K76.5, K76.6, K76.7, K76.8, K76.9, Z94.4, K71.1, K71.3, K71.4, K71.5, K71.7, K76.0, K76.2, I86.4, I98.2 |
| Congestive heart failure | 398.91, 402.01, 402.91, 404.01, 404.03, 404.11, 404.13, 404.91, 404.93, 425.4, 425.5, 425.7, 425.8, 425.9, 428 | I43, I50, I09.9, I11.0, I13.0, I25.5, I42.0, I42.5, I42.6, I42.7, I42.8, I42.9, P29.0 |
| Pulmonary circulation disorders | 416, 415.0, 415.1, 417.0, 417.8, 417.9 | I26, I27, I28 |
| Neurologic disorders | 333.92, 331.9, 332.0, 332.1, 333.4, 333.5, 348.1, 348.3, 780.3, 784.3, 336.2, 334, 335, 340, 341, 345 | G10, G11, G12, G13, G20, G21, G22, G32, G35, G36, G37, G40, G41, R56, G25.4, G25.5, G31.2, G31.8, G31.9, G93.1, G93.4, R47.0 |
|  |  |  |
| **Complications** |  |  |
| Requirement of renal replacement therapy | 39.95 | 3E1M39Z, 5A1D00Z, 5A1D60Z, 5A1D70Z, 5A1D80Z, 5A1D90Z |
| Stroke | 433.01, 433.11, 433.21, 433.31, 433.81, 433.91, 434.01, 434.11, 434.91, 437.0, 437.1, 437.4, 437.5, 437.7, 437.9, 997.01, 997.02, 431, 432.0, 432.1, 432.9, 430 | I63, I67.2, I67.81, I67.82, I67.89, I67.7, I67.5, I67.9, G97.81, G97.82, I97.811, I97.821, I61.9, I62.1, I60.9, I62.9, I62.0, I62.1 |
| Deep vein thrombosis | 451.1, 451.2, 451.81, 451.9, 453.2, 453.40, 453.41, 453.42, 453.8, 453.9 | I82.220, I82.4, I82.6, I82.A1, I82.B1, I82.C1, I82.290, I82.890, I82.91, I80.9, I80.3 |
| Pulmonary embolism | 415.1 | I26 |
| Cardiac arrest | 427.5 | I46.2, I46.8, I46.9 |
| Ventricular tachycardia | 427.1 | I47.2 |
| Ventricular fibrillation | 427.41 | I49.01 |
| Cardiac tamponade | 423.3 | I31.4 |
| Myocardial infarction | 410 | I21 |
| Respiratory failure | 518.81, 518.51, 518.53, 518.84 | J96.00, J96.90, J96.20, J95.821, J95.822 |
| Prolonged mechanical ventilation | 96.72 | 5A1955Z |
| Pneumonia | 480, 481, 482, 483, 485, 486, 997.31, 997.32 | J12, J13, J14, J15, J16, J18, J95.851, J95.89 |
| Pneumothorax | 512.1 | J95.811 |
| Acute respiratory distress syndrome | 518.5, 518.82 | J80, R06.03 |
| Septicemia | 038, 995.91, 995.92, 999.3, 998.51, 998.59 | A40, A41, R65.20, T814XXA, K68.11 |
| Abscess | 569.5, 790.7 | R78.81, K63.0 |
| Wound infection | 998.31, 998.32, 998.5 | T81.32XA, T81.31XA, T81.4XXA, K68.11 |
| Urinary tract infection | 599.0 | N39.0 |
| Hemorrhage | 998.11 | D78.01, D78.02, D78.21, D78.22, E36.01, E36.02, E89.810, E89.811, G97.31, G97.32, G97.51, G97.52, H59.111, H59.112, H59.113, H59.119, H59.121, H59.122, H59.123, H59.129, H59.311, H59.312, H59.313, H59.319, H59.321, H59.322, H59.323, H59.329, H95.21, H95.22, H95.41, H95.42, I97.410, I97.411, I97.418, I97.42, I97.610, I97.611, I97.618, I96.620, J95.61, J95.62, J95.830, J95.831, K91.61, K91.62, K91.840, K91.841, L76.01, L76.02, L76.21, L76.22, M96.810, M96.811, M96.830, M96.831, N99.61, N99.62, N99.820, N99.821 |
| Accidental organ puncture | 998.2 | D78.11, D78.12, E36.11, E36.12, G97.48, G97.49, H59.219, H59.228, H95.31, H95.32, I97.51, I97.52, J95.71, J95.72, K91.71, K91.72, L76.11, L76.12, M96.820, M96.821, N99.71, N99.72, T88.8XXA |
| Phrenic nerve injury | 519.4 | J98.6 |
| Vagus nerve injury | 951.8 | S04.899A |
| Requirement of blood transfusion | 990 | 30233H0, 30233N0, 30243H0, 30243N0, 30253H0, 30253N0, 30263H0, 30263N0, 30233H1, 30243H1, 30253H1, 30263H1, 30233H0, 30233N0, 30233W0, 30243H0, 30243N0, 30243W0, 30253H0, 30253N0, 30253W0, 30233H0, 30233N0, 30233W0, 30243H0, 30263H0, 30263N0, 30263W0, 30233H1, 30243H1, 30253H1, 30263H1, 30233N1, 30233P1, 30243N1, 30243P1, 30253N1, 30253P1, 30263N1, 30263P1, 30233R1, 30243R1, 30253R1, 30263R1, 30233T1, 30233V1, 30233W1, 30243T1, 30243V1, 30243W1, 30253T1, 30253V1, 30253W1, 30263T1, 30263V1, 30263W1, 30233J1, 30233K1, 30233L1, 30233M1, 30243J1, 30243K1, 30243L1, 30243M1, 30253J1, 30253K1, 30253L1, 30253M1, 30263J1, 30263K1, 30263L1, 30263M1, 3E033GC, 3E043GC, 3E053GC, 3E063GC, 30233Q1, 30243Q1, 30253Q1, 30263Q1 |
