# Supplementary material for: National clinical and financial outcomes associated with acute kidney injury following esophagectomy for cancer
Source: PLoS One. 2024 Mar 28;19(3):e0300876. doi: 10.1371/journal.pone.0300876 (PMC10977786; doi:10.1371/journal.pone.0300876)
Supplement: S2 Table — Model C-statistic: 0.79. Ref: Reference. CI: Confidence interval. (DOCX) [file pone.0300876.s002.docx]

Supplemental Table 2. Patient, operative, and hospital characteristics associated with acute kidney injury after esophagectomy for cancer*.* Model C-statistic: 0.79. *Ref: Reference. CI: Confidence interval.*

| **Parameter** | **AOR [95% CI]** | **p-value** |
| --- | --- | --- |
| Age (per year) | 1.01 [1.01-1.02] | 0.002 |
| Female sex | 0.55 [0.45-0.68] | <0.001 |
| *Payer Status* |  |  |
| Private | Ref |  |
| Medicare | 1.11 [0.94-1.32] | 0.20 |
| Medicaid | 1.09 [0.83-1.43] | 0.54 |
| Other | 1.29 [0.92-1.82] | 0.14 |
| Year of admission | 1.12 [1.08-1.15] | <0.001 |
| Minimally invasive approach | 0.80 [0.68-0.95] | 0.01 |
| History of radiation/chemotherapy | 0.52 [0.43-0.63] | <0.001 |
| *Cancer Type (%)* |  | 0.02 |
| Esophageal cancer | Ref |  |
| Gastric cancer | 0.93 [0.81-1.07] | 0.30 |
| *Comorbidities (%)* |  |  |
| Elixhauser Comorbidity Index | 1.08 [1.03-1.14] | 0.001 |
| Diabetes | 1.07 [0.91-1.26] | 0.41 |
| Chronic kidney disease | 2.67 [2.04-3.50] | <0.001 |
| Chronic lung disease | 0.95 [0.81-1.12] | 0.56 |
| Chronic liver disease | 1.52 [1.19-1.94] | 0.001 |
| Congestive heart failure | 1.34 [1.06-1.69] | 0.01 |
| *Hospital Esophagectomy Volume* |  |  |
| Low volume | Ref |  |
| Medium volume | 1.11 [0.78-1.58] | 0.56 |
| High volume | 1.09 [0.78-1.52] | 0.63 |
| *Hospital Teaching Status* |  |  |
| Non-metropolitan | Ref |  |
| Metropolitan non-teaching | 1.85 [0.91-3.79] | 0.09 |
| Metropolitan teaching | 1.54 [0.77-3.06] | 0.22 |
| *Complications* |  |  |
| Cerebrovascular | 1.74 [0.91-3.30] | 0.09 |
| Thromboembolic | 1.21 [0.90-1.64] | 0.20 |
| Cardiac | 1.92 [1.49-2.47] | <0.001 |
| Respiratory | 2.50 [2.16-2.89] | <0.001 |
| Infectious | 3.82 [3.32-4.40] | <0.001 |
| Intraoperative | 0.97 [0.70-1.34] | 0.86 |
| Blood transfusion | 1.23 [1.01-1.51] | 0.04 |
