# Supplementary material for: National clinical and financial outcomes associated with acute kidney injury following esophagectomy for cancer
Source: PLoS One. 2024 Mar 28;19(3):e0300876. doi: 10.1371/journal.pone.0300876 (PMC10977786; doi:10.1371/journal.pone.0300876)
Supplement: S3 Table — Restricted cubic spline knots = 2. CI: Confidence interval. Ref: Reference. (DOCX) [file pone.0300876.s003.docx]

Supplemental Table 3. Royston-Parmar risk-adjusted hazard analysis for 30-day readmission. Restricted cubic spline knots = 2. *CI: Confidence interval. Ref: Reference.*

| **Parameter** | **Exponentiated hazard coefficient [95% CI]** | **p-value** |
| --- | --- | --- |
| Acute kidney injury | 1.16 [1.01-1.32] | 0.03 |
| Age (per year) | 1.00 [1.00-1.01] | 0.07 |
| Female sex | 1.06 [0.96-1.16] | 0.25 |
| *Payer Status* |  |  |
| Private | Ref |  |
| Medicare | 1.06 [0.96-1.17] | 0.24 |
| Medicaid | 0.89 [0.76-1.05] | 0.16 |
| Other | 1.14 [0.91-1.42] | 0.24 |
| Year of admission | 1.00 [0.99-1.01] | 0.97 |
| *Cancer Type (%)* |  | 0.02 |
| Esophageal cancer | Ref |  |
| Gastric cancer | 0.92 [0.85-0.99] | 0.04 |
| *Comorbidities (%)* |  |  |
| Diabetes | 1.13 [1.03-1.23] | 0.009 |
| Hypertension | 1.02 [0.94-1.10] | 0.68 |
| Chronic lung disease | 1.05 [0.96-1.14] | 0.32 |
| Chronic kidney disease (per stage) | 1.10 [0.92-1.31] | 0.31 |
| Congestive heart failure | 1.08 [0.92-1.25] | 0.34 |
| Pulmonary circulation disorders | 0.99 [0.75-1.31] | 0.94 |
| *Hospital Esophagectomy Volume* |  |  |
| Low volume | Ref |  |
| Medium volume | 1.05 [0.81-1.35] | 0.73 |
| High volume | 1.25 [0.98-1.58] | 0.07 |
| *Hospital Teaching Status* |  |  |
| Non-metropolitan | Ref |  |
| Metropolitan non-teaching | 1.12 [0.69-1.81] | 0.64 |
| Metropolitan teaching | 1.28 [0.80-2.03] | 0.30 |
| *Complications* |  |  |
| Cerebrovascular | 0.93 [0.48-1.79] | 0.83 |
| Thromboembolic | 1.18 [0.89-1.57] | 0.25 |
| Cardiac | 0.80 [0.64-1.01] | 0.06 |
| Respiratory | 1.06 [0.97-1.16] | 0.17 |
| Infectious | 0.94 [0.85-1.05] | 0.28 |
| Intraoperative | 1.00 [0.80-1.24] | 0.98 |
| Blood transfusion | 1.05 [0.95-1.17] | 0.34 |
